# Liver adrenoceptor alpha-1b plays a key role in energy and glucose homeostasis in female mice

**DOI:** 10.1152/ajpendo.00153.2024

**Published:** 2024-09-11

**Authors:** Anisia Silva, Mathilde Mouchiroud, Olivier Lavoie, Sarra Beji, Joel K. Elmquist, Alexandre Caron

**Affiliations:** ^1^Faculty of Pharmacy, Université Laval, Quebec City, Quebec, Canada; ^2^Quebec Heart and Lung Institute, Quebec City, Quebec, Canada; ^3^Department of Internal Medicine, Center for Hypothalamic Research , University of Texas Southwestern Medical Center, Dallas, Texas, United States

**Keywords:** adrenergic receptor, glucose metabolism, liver, sex difference, sympathetic nervous system

## Abstract

The liver plays a major role in glucose and lipid homeostasis and acts as a key organ in the pathophysiology of metabolic diseases. Intriguingly, increased sympathetic nervous system (SNS) activity to the liver has been associated with the development and progression of type 2 diabetes and obesity. However, the precise mechanisms by which the SNS regulates hepatic metabolism remain to be defined. Although liver α1-adrenoceptors were suggested to play a role in glucose homeostasis, the specific subtypes involved are unknown mainly because of the limitations of pharmacological tools. Here, we generated and validated a novel mouse model allowing tissue-specific deletion of α-1b adrenoceptor (*Adra1b*) in hepatocytes to investigate the role of liver ADRA1B in energy and glucose metabolism. We found that selective deletion of *Adra1b* in mouse liver has limited metabolic impact in lean mice. However, loss of *Adra1b* in hepatocytes exacerbated diet-induced obesity, insulin resistance, and glucose intolerance in female, but not in male mice. In obese females, this was accompanied by reduced hepatic gluconeogenic capacity and reprogramming of gonadal adipose tissue with hyperleptinemia. Our data highlight sex-dependent mechanisms by which the SNS regulates energy and glucose homeostasis through liver ADRA1B.

**NEW & NOTEWORTHY** The sympathetic nervous system plays an important role in regulating hepatic physiology and metabolism. However, the identity of the adrenoceptors involved in these effects is still elusive. Using CRISPR-Cas9, we developed a novel transgenic tool to study the role of liver α-1b adrenoceptor (ADRA1B). We show that ADRA1B plays a key role in mediating the effects of the sympathetic nervous system on hepatic metabolism, particularly in female mice.

## Graphical abstract

**Figure:**
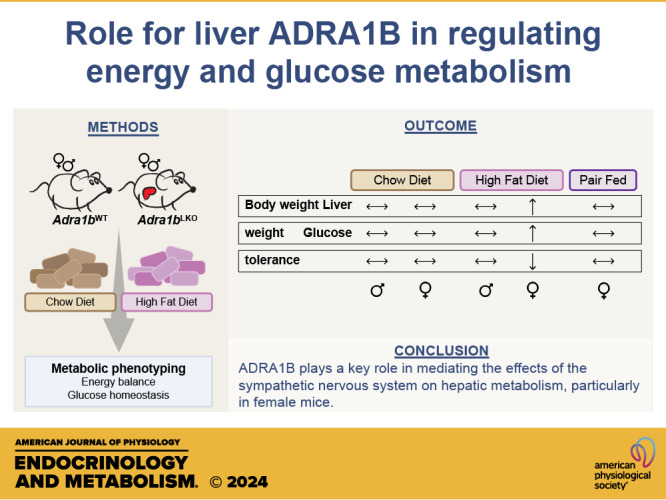

## INTRODUCTION

The prevalence of insulin resistance and type 2 diabetes (T2D) is rising at an alarming rate worldwide. Data from the 10th edition of the International Diabetes Federation Atlas (IDF; 1) show that 537 million adults are currently living with diabetes (10.5% of the population) and predict this number to jump to 783 million (12.2%) by 2045. Therefore, there is an urgent need to better understand the pathophysiology of this disease, which could help to identify new strategies to prevent the deleterious impact of chronic hyperglycemia.

The liver plays major roles in glucose and lipid homeostasis and acts as a key organ in the pathophysiology of metabolic diseases. Hepatic insulin resistance is associated with increased hepatic glucose production, contributing to chronic hyperglycemia. Furthermore, damaged livers release cytokines and hepatokines known to contribute to dyslipidemia, atherosclerosis, and alterations of other organs (2, 3). Intriguingly, increased sympathetic nervous system (SNS) activity and decreased parasympathetic nervous system (PNS) activity have both been associated with the development and progression of T2D and obesity (4, 5). Furthermore, evidence suggests that obesity and T2D may first lead to increased sympathetic outflow to the liver (compensation), followed by a progressive decrease in the number of hepatic sympathetic fibers (neuropathy) (6–10). However, the precise mechanisms by which the SNS regulates hepatic metabolism remain to be defined.

The monoamine noradrenaline (NE) is the main neurotransmitter released by the sympathetic nerves (11). NE binds to nine different adrenoceptor subtypes, which are widely expressed in different brain regions and peripheral organs where they mediate the effects of catecholamines on cardiovascular, reproductive, and metabolic functions (12). In the liver, adrenoceptors expressed on hepatocytes modulate several aspects of metabolism, including hepatic glucose production and fatty acid metabolism (13). Injection of NE rapidly mobilizes glucose from the liver by increasing both glycogenolysis and gluconeogenesis (14). This hyperglycemic effect is suggested to be mediated, at least in part, by liver α1-adrenoceptors (14, 15). Although α1-adrenoceptors are known to be important for glucose homeostasis (16, 17), the specific subtype involved in this process is unknown because of the limitations of pharmacological tools. One reasonable subtype candidate is the α-1b adrenoceptor (ADRA1B). Mice lacking *Adra1b* globally are hyperinsulinemic and exhibit high hepatic glycogen content, as well as impaired liver insulin sensitivity (18). However, confounding factors resulting from the global deletion make the direct contribution of liver ADRA1B to glucose homeostasis hard to dissect. This includes the fact that these mice are hyperleptinemic and have increased parasympathetic activity at the level of the pancreas, which could affect insulin levels (18). Moreover, *Adra1b* is expressed in many brain nuclei known to regulate peripheral glucose metabolism and liver homeostasis (19), and targeted disruption of the mouse *Adra1b* gene causes cardiac defects and hypofertility (20–23). Therefore, the development of a liver-specific *Adra1b* knockout (KO) model is needed to determine the direct role of hepatic ADRA1B signaling in the control of glucose homeostasis.

Here, we developed a conditional mouse model for the *Adra1b* gene and generated mice lacking *Adra1b* specifically in hepatocytes to directly investigate the role of liver ADRA1B in energy and glucose metabolism. We found that selective deletion of *Adra1b* in mouse liver has limited metabolic impact in lean mice. However, loss of *Adra1b* in hepatocytes exacerbated diet-induced obesity, insulin resistance, and glucose intolerance in female, but not in male mice. In obese females, this was accompanied by reduced hepatic gluconeogenic capacity and reprogramming of gonadal adipose tissue. Our data highlight sex-dependent mechanisms by which the SNS regulates liver metabolism through ADRA1B.

## MATERIALS AND METHODS

### Animals

Animal care and handling were performed in accordance with the Canadian Guide for the Care and Use of Laboratory Animals. All experimental procedures received prior approval from the Laval University Animal Care Committee (CPAUL). Mice were maintained on a 12-h light/dark cycle (lights on 0600–1800), while group-housed in ventilated cages at an ambient temperature of 23 ± 1°C. All mice were maintained on a C57BL/6J background and were fed ad libitum a chow diet (Harlan Teklad, 2918) prior to the experimental procedures. For diet-induced obesity studies, mice were fed a high-fat diet (45% kcal fat, Research Diets, D12451) for 8 wk. Male and female mice were aged 8–12 wk at the beginning of the experiments.

### Generation of *Adra1b^LKO^* Mice

Conditional mouse model for the *Adra1b* gene was developed using Alt-R CRISPR-Cas9 System from Integrated DNA Technologies. Briefly, two synthetic sgRNA targeting introns 2 and 5 (crRNA 1: 5′-GGG GAA CTA AAG TAT ACG CCT GG-3′; crRNA 2: 5′-GAG GGG AGG TAG ACC TAC ATT GG-3′) were synthetized and co-injected with the tracrRNA, two Ultramer DNA Oligonucleotides containing the LoxP sequence and ∼60 bp of homology arm, and Alt-R S.p Cas9 Nuclease 3NLS, in C57BL6 ES cells by the UTSW Transgenic Core. Mice were screened and the founder backcrossed with a C57BL6 mouse to ensure germline transmission. The heterozygous (Adra1b^fl/+^) F1 pups were then bred with an *Albumin*-Cre mouse (developed by Mark A. Magnuson, JAX Stock No.: 003574) and the F2 pups *Albumin*-Cre^+/−^::Adra1b^fl/+^ bred with heterozygous (Adra1b^fl/+^) F1 pups to generate mice lacking *Adra1b* specifically in liver (*Albumin*-Cre^+/−^::Adra1b^fl/fl^, from now on referred to as *Adra1b*^LKO^). The following primers were used to genotype the animals: forward: 5′-TGT TGG CTC CCC TTC TTC AT-3′; reverse: 5′-CCA AAC ACA CAC TGA TGG CA-3′.

### Glucose Homeostasis

For the glucose tolerance tests (GTT), mice were fasted for 6 h and were injected intraperitoneally with 1 g/kg of d-Glucose (Sigma Aldrich, St Louis, MO). For the insulin tolerance test (ITT), mice were fasted for 4 h and were injected intraperitoneally with 0.75 U/kg of insulin (Humulin, Lilly, Canada). Blood glucose from the tail vein at different points time was measured using a glucometer (OneTouch). The homeostasis model assessment of insulin resistance (HOMA-IR) index was calculated in 4-h fasted mice based on the following formula: fasting insulinemia (μUI/mL) × fasting glycemia (mM) ÷ 22.5.

### Metabolic Cage Experiments

Oxygen consumption (V̇o_2_), energy expenditure (EE), respiratory exchange ratio (RER), food intake, and water intake were evaluated by the Quebec Mouse Phenotyping Core using a 16-cage Promethion core system (Sable Systems International, Las Vegas, NV) located in a temperature- and light-controlled cabinet. Female mice were fed a high-fat diet (45% kcal fat, Research Diets, D12451) for 8 wk while being individually housed. Mice were then housed for 72 h (acclimation) before measurements were performed for 72 h. Gas analyzers, food, and water precision mass monitors were calibrated just before the experiment. Acquired data were processed using ExpeData and Macro Interpreter (v.23.) running Macro v.2.52.0-slice5min.

### Pair-Feeding Experiment

Littermate control mice were provided with a fresh pellet of high-fat diet (45% kcal fat, Research Diets, D12451) at 0900 (ZT3) every morning for 7 wk. Food intake was measured every day and the equivalent amount given to *Adra1b*^LKO^ mice so that they received the average amount of food consumed by littermate controls the day before.

### Triglyceride, Cholesterol, and Glycogen Extraction and Measurement

For triglyceride and cholesterol analysis in tissue, lipids were extracted as described by Folch et al. (24). Lipids were suspended in isopropanol and quantified using commercial assay kits (RANDOX, TR210 and CH200). Glycogen was measured in liver samples as described previously (25). In brief, glycogen was extracted in 30% KOH saturated with Na_2_SO_4_, precipitated in 95% ethanol, and resuspended in distilled H_2_O. After the addition of phenol and H_2_SO_4_, absorbance at 490 nm was measured in triplicates. The glycogen concentration was calculated using a standard curve from commercial glycogen (Millipore Sigma, 10901393001).

### Liver Histology

Liver tissue samples were fixed for 48 h in 10% formalin at 4°C. Tissues were next dehydrated, embedded in paraffin, and cut into 10-µm thick sections. Sections were stained with hematoxylin and eosin (H&E). All pictures were taken using an Olympus BX51 bright-field microscope equipped with a Retiga 2000R Fast 1394 camera. Image acquisition was performed with the Image-Pro plus program.

### Plasma Measurements

Blood was collected through cardiac puncture using syringes conditioned with EDTA. Plasma was prepared from blood samples and stored at −80°C. Hormones and metabolites were measured according to the manufacturer’s instructions using the following commercial kits: leptin (Crystal Chem, 90030), insulin (Crystal Chem, 90080), triglycerides (RANDOX, TR210), and cholesterol (RANDOX, CH200).

### Quantitative Real-Time PCR

Total mRNA was isolated from liver, hypothalamus, heart, and gonadal white adipose tissue (gWAT) using TRI Reagent (Millipore Sigma T9424). The RNA concentrations were estimated from absorbance at 260 nm and cDNA synthesis was performed using qScript cDNA SuperMix (Qantabio 95048-500). mRNA extraction and cDNA synthesis were performed following the manufacturer’s instructions. cDNA was diluted in DNase-free water (1:15) before the quantification by real-time PCR. mRNA transcript levels were measured in duplicate samples using a CFX384 Touch Real-Time PCR system (Bio-Rad). Chemical detection of the PCR products was achieved with PerfeCTa SYBR Green FastMix (Qantabio, 95071-05K). Gene expression was normalized to the expression level of reference genes. Fold differences in target mRNA expression were measured using the 2Δ-cycle threshold method by comparison with the housekeeping genes (*Rn18s*, *Rplp0*, and *B2m*) and expressed as fold change versus littermate controls. The sequences of the primers used are shown in Supplemental Table S1.

### Chromogenic In Situ Hybridization

Liver samples were fixed for 48 h in 10% formalin at 4°C. Tissues were next dehydrated, embedded in paraffin, cut into 10-µm thick sections and mounted on Superfrost Plus microscope slides (Thermo Fisher Scientific) and then kept at room temperature. All chromogenic in situ hybridization experiments were performed using the RNAscope 2.5 HD Duplex Detection kit (Advanced Cell Diagnostics) with slight modifications to the manufacturer’s protocol. Briefly, Formalin-Fixed Paraffin-Embedded (FFPE) liver sections were first deparaffinized and the slides were incubated at 60°C for 60 min in ACD HybEz^TM^ II Oven. Sections were then incubated in xylene and in 100% ethanol (EtOH) baths. Samples were subsequently treated for 10 min at room temperature with H_2_O_2_, immerged in 1× Target Retrieval Reagent (Advanced Cell Diagnostics) for 30 min at ∼96°C, rinsed with fresh water, and dried out in 100% EtOH for 3 min. An ImmEdge Hydrophobic Barrier Pen (Vector Laboratories) was then used to draw a hydrophobic barrier around liver sections, and samples were incubated with Protease Plus (Advanced Cell Diagnostics) for 30 min at 40°C. Following this step, slides were rinsed with fresh water and incubated with a probe mix containing Mm-*Adra1b*-C1 (413561) for 2 h at 40°C. After incubation, slides were washed in 1× Wash Buffer (Advanced Cell Diagnostics) and kept overnight in 5× saline sodium citrate buffer (SSC) at room temperature. All of the following incubation steps were performed according to the manufacturer’s protocol. Slides were incubated with AMP1 reagent for 30 min, AMP2 for 15 min, AMP3 for 30 min, and AMP4 for 15 min at 40°C. Slides were then incubated with AMP5 for 30 min and AMP6 for 15 min at room temperature and subsequently with AMP7 for 15 min and AMP8 for 30 min at 40°C. Finally, slides were incubated with AMP9 for 30 min and AMP10 for 15 min at room temperature. C1 probe signal was then revealed for 10 min at room temperature, and liver sections were counterstained using 50% Gill’s hematoxylin and 0.02% ammonia water. Slides were coverslipped using VectaMount permanent mounting medium (H-5600; Vector Laboratories) and imaged using a Zeiss AxioScan.Z1 microscope.

### Data Analysis

Values are presented as means ± SE. Statistical analyses were performed using GraphPad Prism 10. When comparing two groups, significance was determined by two-tailed, unpaired *t* test. Experiments including multiple groups and factors were analyzed using two-way ANOVA with Sidak’s multiple-comparisons test. *P* value <0.05 was considered statistically significant.

## RESULTS

### Generation of Liver-Specific *Adra1b* Knockout Mice

**Figure 1.**
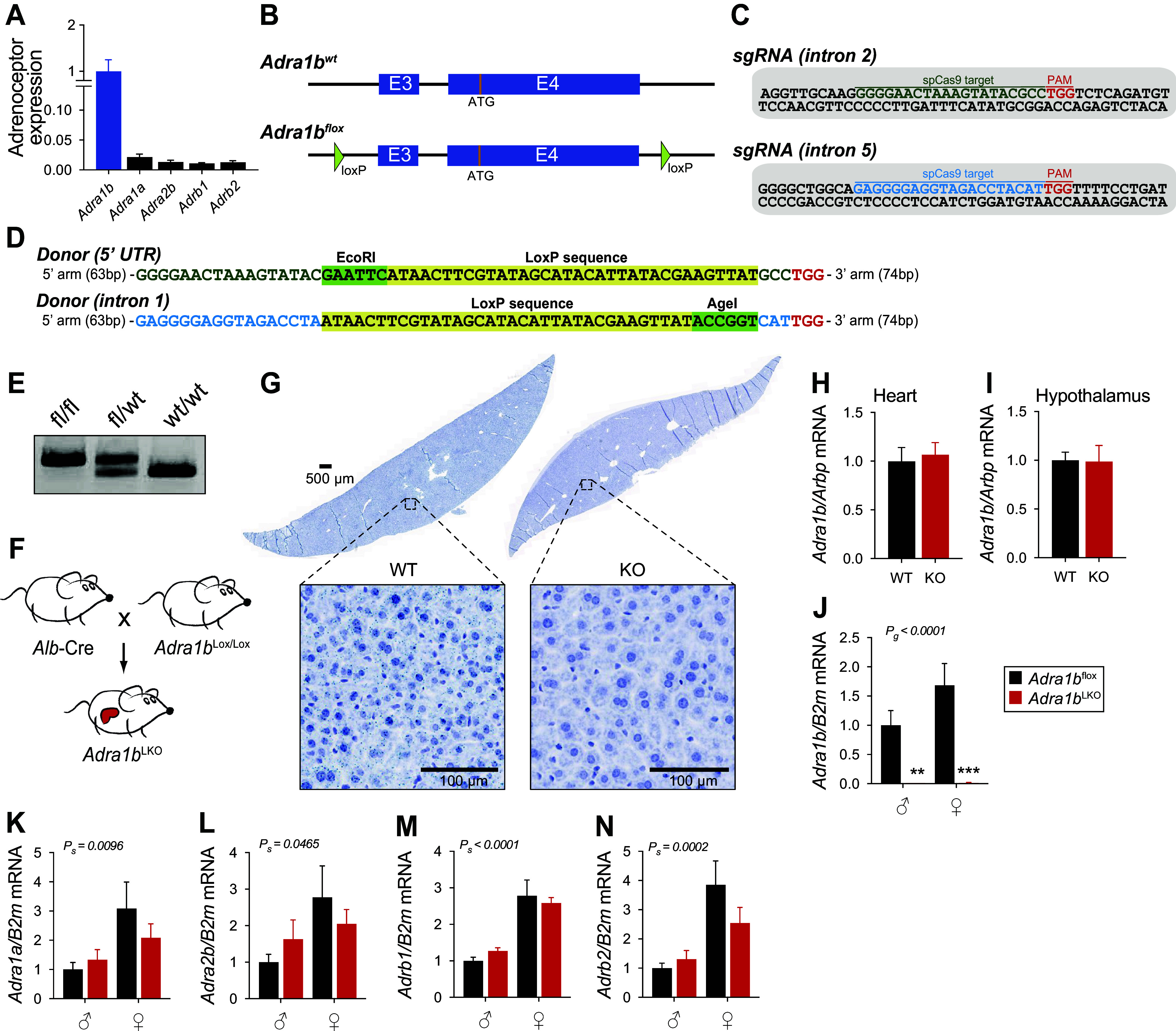
Generation and validation of liver-specific *Adra1b* knock-out mice. *A*: gene expression analysis of the subtypes of adrenoceptors detected in the liver of C57BL/6J mice (*n* = 8). *B*: using CRISPR-Cas9 technology, exon 3 and exon 4 (which contains the start codon) of the *Adra1b* gene were targeted and flanked by two loxP sites. *C*: sequences of the two synthetic sgRNA synthetized to insert the LoxP sites. *D*: sequences of the donor templates consisting of 200 bp ultramer DNA oligonucleotides. *E*: genotyping of *Adra1b*^fl/fl^, *Adra1b*^fl/+^, and *Adra1b*^+/+^ mice. *F*: schematic representation of the breeding strategy used to generate *Adra1b*^LKO^ mice. *G*: chromogenic in situ hybridization showing the presence in *Adra1b*^WT^ and the absence in *Adra1b*^LKO^, of *Adra1b* (blue dots). Representative image from *n* = 2 mice/group. Expression of *Adra1b* in heart (*H*) and hypothalamus (*I*) of *Adra1b*^WT^ and *Adra1b*^LKO^ mice (*n* = 8/group). Expression of *Adra1b* (*J*), *Adra1a* (*K*), *Adra2b* (*L*), *Adrb1* (*M*), and *Adrb2* (*N*) in the liver of male and female *Adra1b*^WT^ and *Adra1b*^LKO^ mice (*n* = 6–8/group). Significant differences (*P* < 0.05) between groups (assessed by two-way ANOVA) are presented in each graph (**P < 0.01, ***P < 0.0001). KO, knockout; WT, wild type.

Confirming previous studies (26, 27), *Adra1b* is the most highly expressed adrenoceptor subtype in the mouse liver (Fig. 1*A*). To better understand the function of liver ADRA1B, we generated and validated a novel mouse model allowing tissue-specific deletion of *Adra1b*. In brief, exons 3 and 4 of the *Adra1b* gene were floxed using CRISPR-Cas9 (Fig. 1*B*). The sequences of the two synthetic sgRNA and oligonucleotides containing the LoxP sites used are presented in Fig. 1, *C* and *D*. Heterozygous (*Adra1b*^fl/+^) mice (see genotyping data in Fig. 1*E*) were bred with *Albumin(Alb)*-Cre mice (JAX Stock No: 003574, Fig. 1*F*) and the resulting pups (*Alb*-Cre^+/−^::*Adra1b*^fl/+^) bred with *Adra1b*^fl/fl^ mice to generate mice lacking *Adra1b* specifically in liver (*Alb*-Cre^+/−^::*Adra1b*^fl/fl^, from now on referred to as *Adra1b*^LKO^). RNAScope in situ hybridization confirmed the absence of *Adra1b* signal in hepatocytes of *Adra1b*^LKO^ mice (Fig. 1*G*). Confirming the specificity of the deletion in the liver, *Adra1b* expression was not impaired in organs expressing high levels of it such as the heart and hypothalamus (Fig. 1, *H* and *I*). The loss of *Adra1b* in the liver did not lead to compensatory increase of other adrenoceptors (Fig. 1, *J*–*N*). These results validate a novel mouse model allowing targeted deletion of *Adra1b* in a Cre-dependent manner.

### Loss of Liver *Adra1b* Has Limited Metabolic Impact in Lean Mice

**Figure 2.**
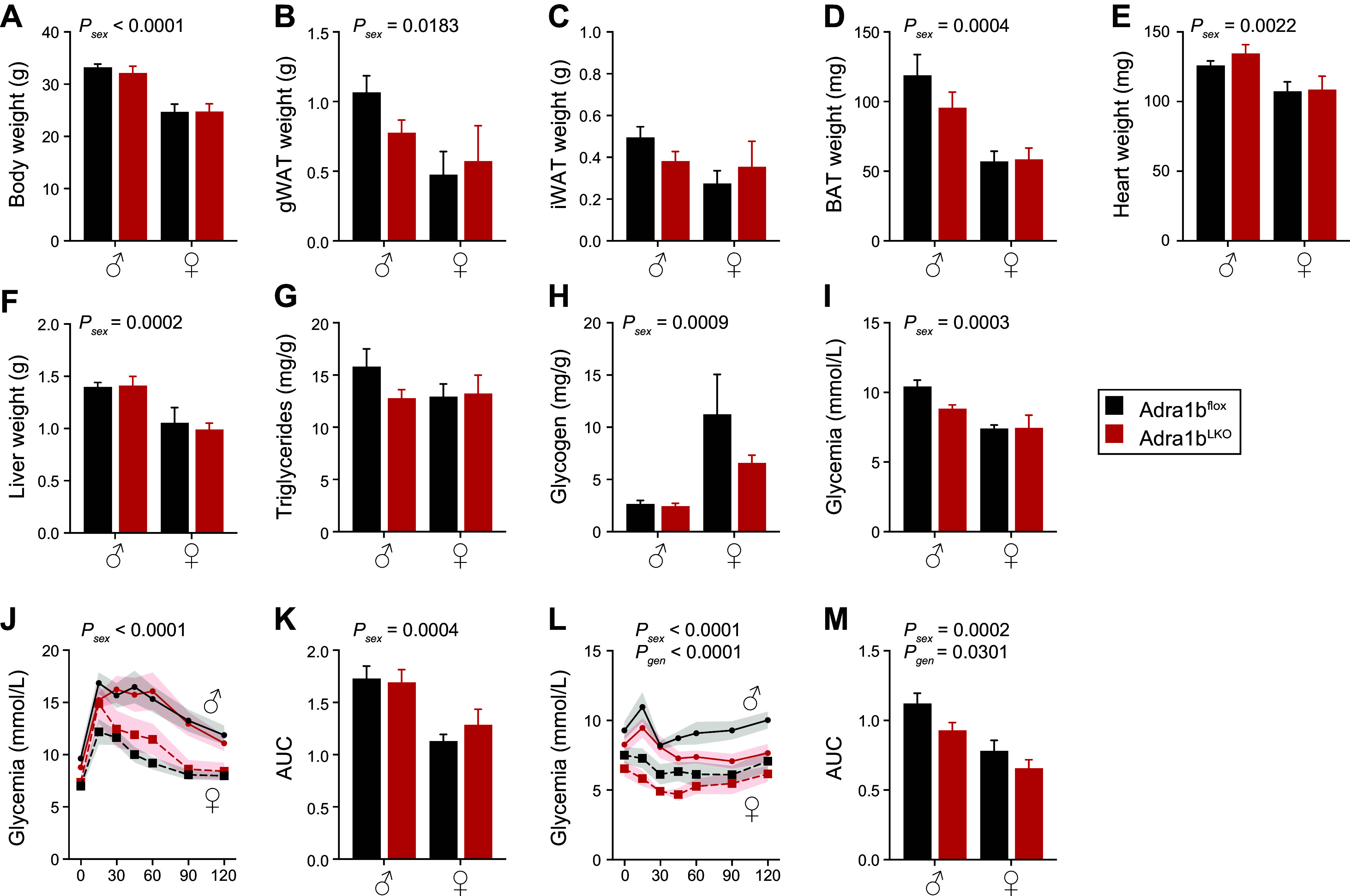
Loss of liver *Adra1b* has limited impact in male and female mice fed laboratory chow. *A*: body weight of 24-wk-old male (*n* = 8/group) and female (*n* = 6/group) *Adra1b*^WT^ and *Adra1b*^LKO^ mice fed laboratory chow. Weight of gonadal white adipose tissue (gWAT, *B*), inguinal (iWAT, *C*), brown adipose tissue (BAT, *D*), heart (*E*), and liver (*F*) of mice presented in *A*. Liver triglyceride (*G*) and glycogen (*H*) content (normalized on liver weight) of mice presented in *A*. *I*: fasting (4 h) glycemia of mice presented in *A*. *J*: glucose tolerance test (GTT) was performed 2 wk before euthanizing the mice. *K*: area under the curve for the GTT shown in *J*. *L*: insulin tolerance test (ITT) was performed 1 wk before euthanizing the mice. *M*: area under the curve for the ITT shown in *L*. Significant differences (*P* < 0.05) between sex and genotype (assessed by two-way ANOVA) are presented in each graph.

Even though the expected sex differences were observed in body weight and weight of adipose, heart, and liver, the absence of *Adra1b* in the liver did not result in any obvious phenotype in both male and female *Adra1b*^LKO^ mice fed with rodent laboratory chow diet compared with littermate controls (Fig. 2, *A–F* and Supplemental Fig. S1*A*). Likewise, liver triglyceride and glycogen contents were similar between genotypes (Fig. 2, *G* and *H*). As expected, female mice had lower fasting glycemia compared with male, but no significant difference was observed between genotypes (Fig. 2*I*). Glucose tolerance was also better in females compared with males with no genotype effect (Fig. 2, *J* and *K*). Interestingly, mice lacking *Adra1b* specifically in the liver had improved insulin sensitivity (Fig. 2, *L* and *M*). These data suggest that removing *Adra1b* from hepatocytes has no substantial effect on energy homeostasis but may play a role in glucose metabolism.

### Female *Adra1b*^LKO^ Are More Susceptible to Diet-Induced Obesity

**Figure 3.**
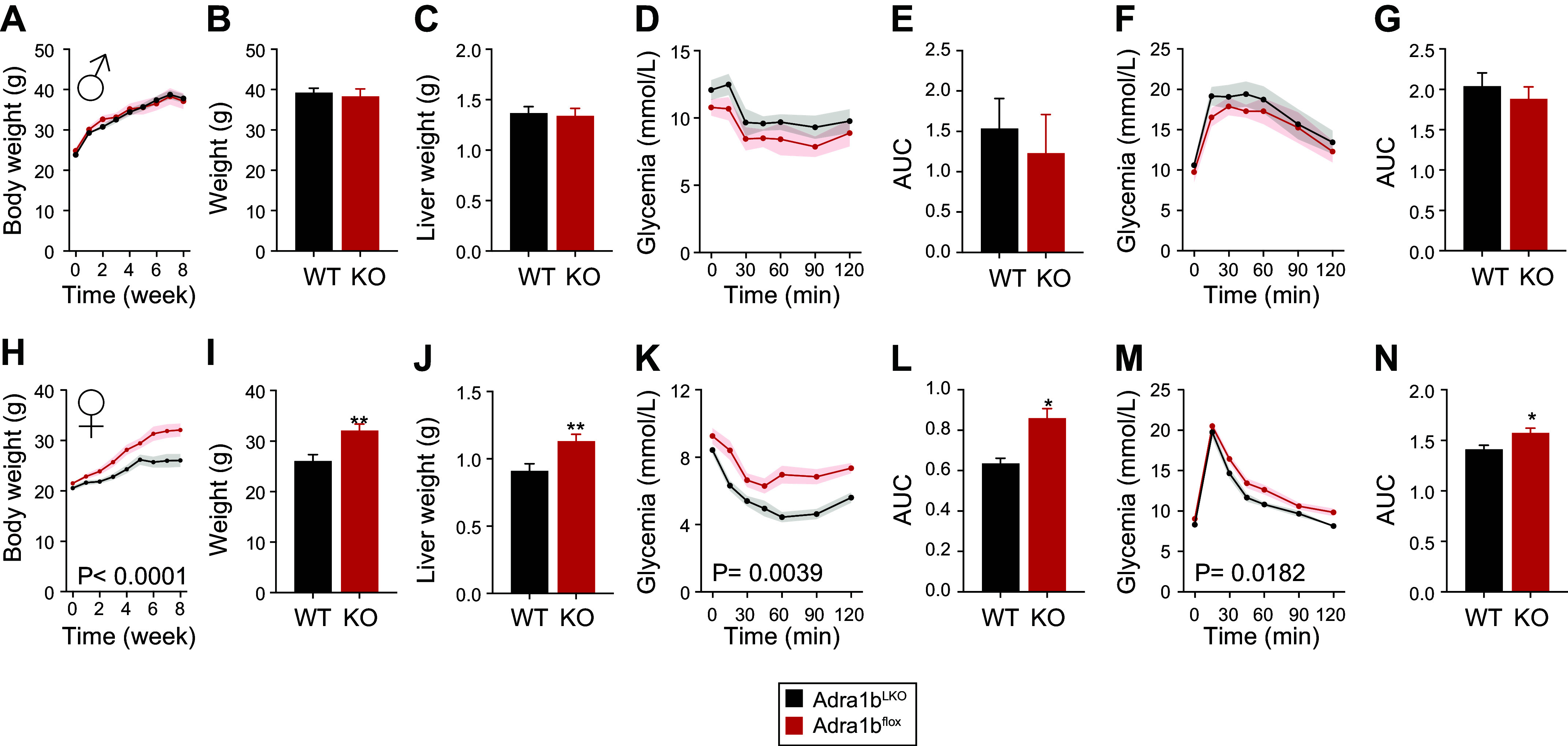
Loss of liver *Adra1b* exacerbates diet-induced obesity, insulin resistance, and glucose intolerance in female, but not in male mice, fed an obesogenic diet. Body weight curve (*A*) and final body weight (*B*) of male *Adra1b*^WT^ and *Adra1b*^LKO^ mice fed a high-fat diet from 10 to 11 wk of age for 8 wk (*n* = 5–8/group). *C*: liver weight of mice presented in *A* and *B*. *D*: glucose tolerance test (GTT) was performed 2 wk before euthanizing the mice. *E*: area under the curve for the GTT shown in *D*. *F*: insulin tolerance test (ITT) was performed 1 wk before euthanizing the mice. *G*: area under the curve for the ITT shown in *L*. Body weight curve (*H*) and final body weight (*I*) of female *Adra1b*^WT^ and *Adra1b*^LKO^ mice fed a high-fat diet from 10 to 11 wk of age for 8 wk (*n* = 9–15/group). *J*: liver weight of mice presented in *H* and *I*. *K*: glucose tolerance test (GTT) was performed 2 wk before euthanizing the mice. *L*: area under the curve for the GTT shown in *K*. *M*: insulin tolerance test (ITT) was performed 1 wk before euthanizing the mice. *N*: area under the curve for the ITT shown in *M*. Significant differences (*P* < 0.05) between groups (assessed by Student’s *t* test for *I*, *J*, *L*, and *N*; by one-way ANOVA for *H*, *K*, and *N*) are presented in each graph. (*P < 0.05, **P < 0.01).

**Figure 4.**
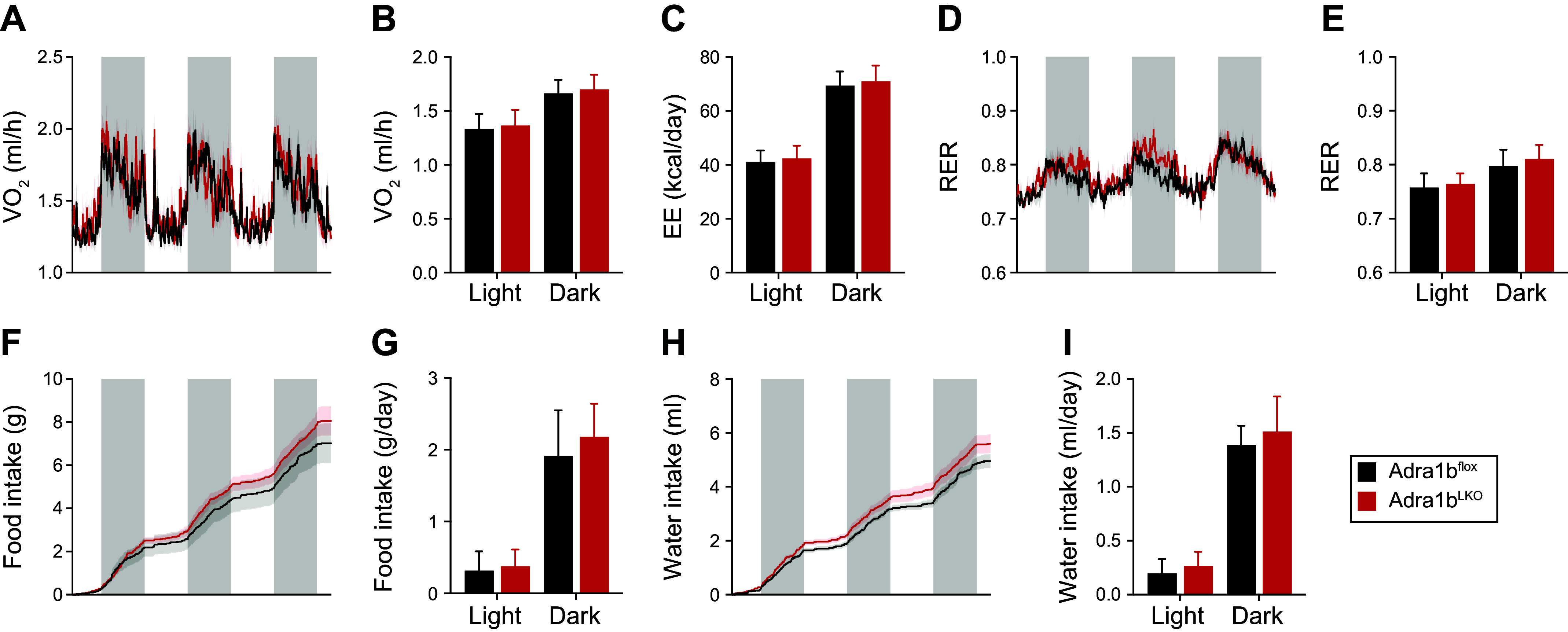
Loss of liver *Adra1b* in obese female mice does not impact energy expenditure. Oxygen consumption (V̇o_2_, *A*) and averageV̇o_2_ (*B*) over 3 days. *C*: average energy expenditure (EE) over 3 days. Respiratory exchange ratio (RER, *D*) and average RER (*E*) over 3 days. Food intake over 3 days (*F*) and average food intake per day (*G*). Water intake over 3 days (*H*) and average water intake per day (*I*) (*n* = 8/group).

**Figure 5.**
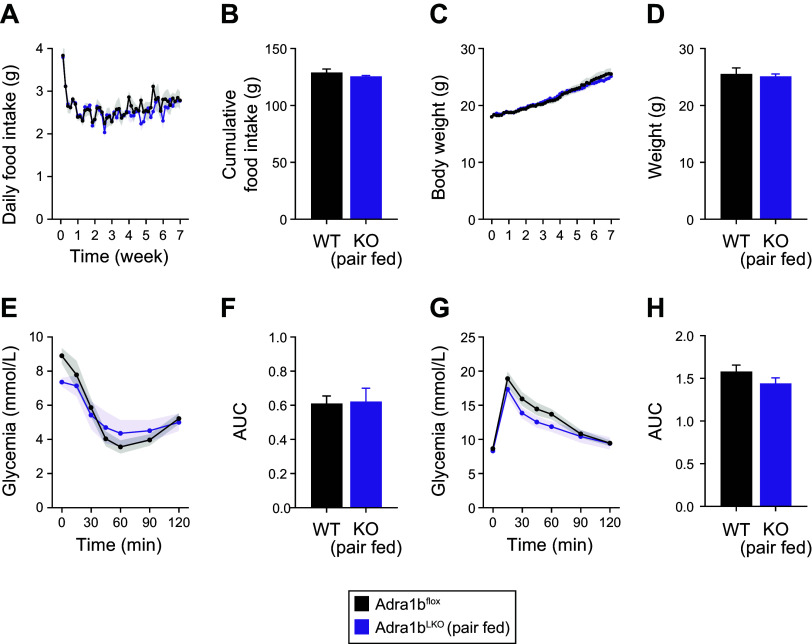
Pair feeding female *Adra1b*^LKO^ mice prevents the exacerbation of obesity and glucose impairments. Daily food intake (*A*) and cumulative food intake (*B*) of female *Adra1b*^WT^ and *Adra1b*^LKO^ mice. The amount of food given to *Adra1b*^LKO^ was based on the average eaten by *Adra1b*^WT^ the day before. Body weight curve (*C*) and body weight (*D*) of *Adra1b*^WT^ and pair-fed *Adra1b*^LKO^ mice. *E*: glucose tolerance test (GTT). *F*: area under the curve for the GTT shown in *E*. *G*: insulin tolerance test (ITT). *H*: area under the curve for the ITT shown in *G* (*n* = 7 or 8/group).

Given these slight changes in glucose metabolism, we next sought to determine the metabolic role of liver ADRA1B in a context of diet-induced obesity (DIO). As shown in Fig. 3, *A–C* and Supplemental Fig. S1*B*, loss of *Adra1b* in the liver of male mice had no impact on body weight and liver weight. Likewise, male *Adra1b*^LKO^ mice had similar insulin sensitivity (Fig. 3, *D* and *E*) and glucose tolerance (Fig. 3, *F* and *G*) compared with littermate controls. On the other hand, female *Adra1b*^LKO^ mice were more susceptible to DIO compared with littermate controls (Fig. 3, *H* and *I*). This was accompanied by increased weight of liver in female mice fed an obesogenic diet (Fig. 3*J*). This effect was however absent after normalizing liver weight on body weight (Supplemental Fig. S1*B*). Female *Adra1b*^LKO^ mice were more insulin resistant and glucose intolerant (Fig. 3, *K*–*N*), despite no difference in insulin levels (1.524 ± 0.467 vs. 1.433 ± 0.2998, *P* = 0.8652) and HOMA-IR (15.22 ± 6.70 vs. 16.02 ± 3.60, *P* = 0.8945). To gain insight into the potential mechanisms driving this obese phenotype, we performed metabolic cages analysis in mice fed an obesogenic diet for 8 wk. No difference between genotype was observed on oxygen consumption, energy expenditure, and RER (Fig. 4, *A–E*). Although no significant change in food and water intake was observed over the recording period (Fig. 4, *F–I*), cumulative food intake trended to be higher in female *Adra1b*^LKO^ mice, suggesting that subtle daily increases in food intake over the 8-wk period could explain the obese phenotype. To confirm whether increased food intake may be driving the obese phenotype in female *Adra1b*^LKO^ mice, we performed a pair-feeding experiment in which *Adra1b*^LKO^ mice were given the average amount of food consumed by littermate controls. Figure 5, *A* and *B*, confirms our approach to normalize food intake between groups. Strikingly, body weight (Fig. 5, *C* and *D*), insulin sensitivity (Fig. 5, *E* and *F*), and glucose tolerance (Fig. 5, *G* and *H*) were no longer impaired in pair-fed *Adra1b*^LKO^ mice. Together, these results indicate that loss of *Adra1b* in the liver exacerbates DIO and impairments in glucose homeostasis in female, but not in male, mice. Furthermore, our results indicate that this phenotype emerges from hyperphagia.

### Female *Adra1b*^LKO^ Have Reduced Hepatic Gluconeogenic Capacity and Exhibit Reprogramming of Gonadal Adipose Tissue

**Figure 6.**
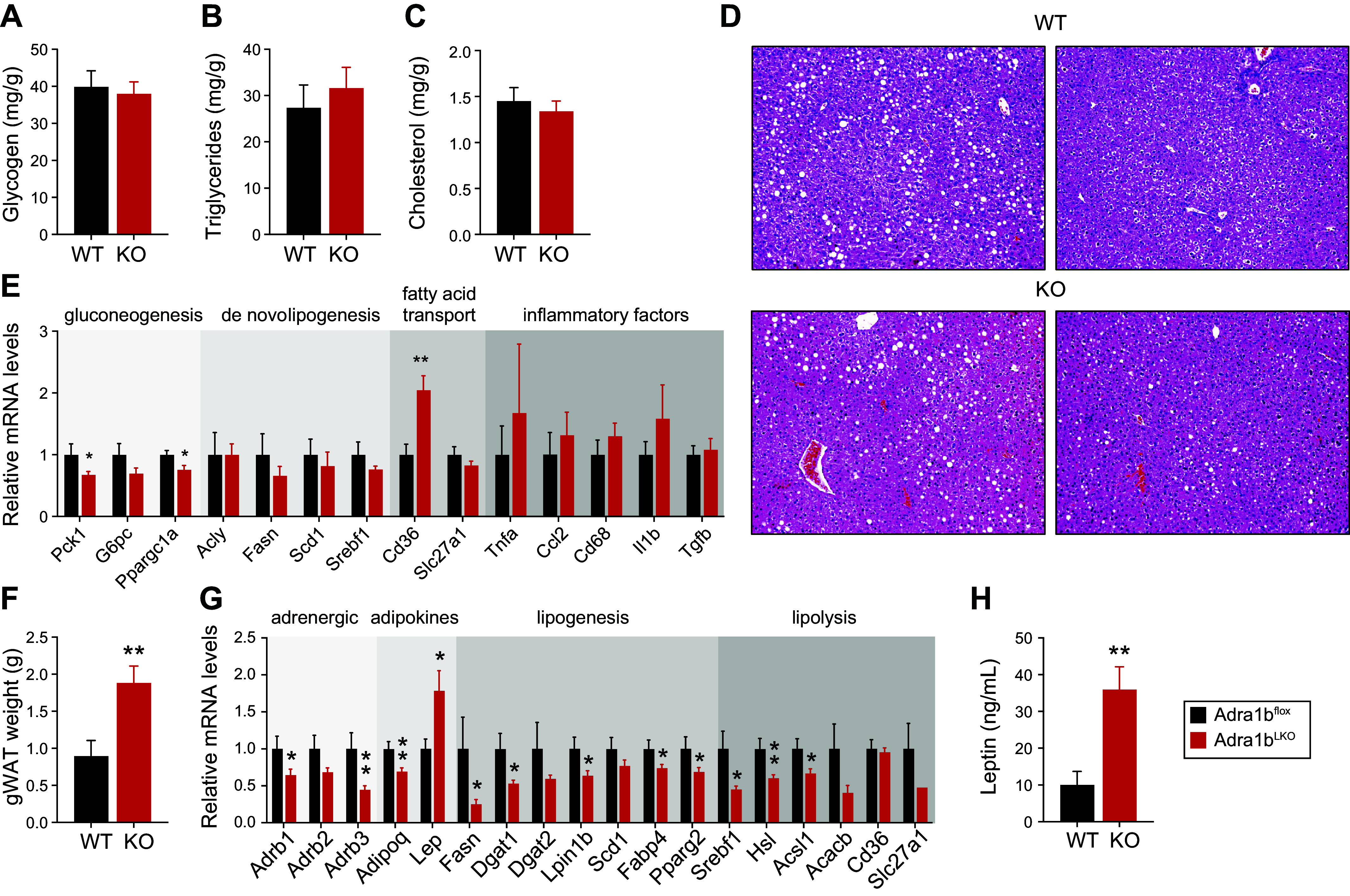
Loss of liver *Adra1b* affects hepatic gluconeogenic capacity and impairs gonadal white adipose tissue in female mice fed an obesogenic diet. Glycogen (*A*), triglyceride (*B*), and cholesterol (*C*) content in the liver of mice presented in Fig. 3. Data are normalized on liver weight. *D*: representative hematoxylin and eosin (H&E) staining of the liver of mice presented in Fig. 3. *E*: analysis of genes involved in gluconeogenesis, de novo lipogeneses, fatty acid transport, and inflammation in the liver. *F*: weight of gonadal white adipose tissue (gWAT). *G*: analysis of the expression of genes coding for adrenergic receptors and adipokines, and of genes involved in lipogenesis and lipolysis in gonadal white adipose tissue of mice presented in Fig. 3. *H*: circulating leptin levels in female *Adra1b*^WT^ and *Adra1b*^LKO^ mice. Significant differences (*P* < 0.05) between groups (assessed by Student’s *t* test) are presented in each graph (*n* = 9–15/group). (*P < 0.05, **P < 0.01).

Given that female *Adra1b*^LKO^ mice were more susceptible to DIO, we next focused our attention on the liver. Female *Adra1b*^LKO^ mice did not show higher levels of hepatic glycogen, triglycerides, or cholesterol (Fig. 6, *A–C*). Likewise, liver histology did not reveal any noticeable changes between genotypes (Fig. 6*D*). Gene expression analysis in the liver revealed a significant reduction in gluconeogenic genes such as *Pck1* and *Ppargc1a*, and an increase in the fatty acid transporter *Cd36* mRNA (Fig. 6*E*). No significant change in the expression of lipogenic and inflammatory gene markers was observed. Supporting the obese phenotype (Fig. 3, *H–I*), gonadal white adipose tissue (gWAT) was significantly bigger in female *Adra1b*^LKO^ mice whether it was normalized or not on body weight (Fig. 6*F* and Supplemental Fig. S1*C*). We also observed a significant decrease in the expression of β-adrenoceptors (*Adrb1* and *Adrb3*) in gWAT, together with a decrease in *Adipoq* expression and an increase in *Lep* expression in *Adra1b*^LKO^ mice (Fig. 6*G*). Expression of genes involved in lipid metabolism was also significantly lower in gWAT of female mice lacking *Adra1b* in the liver (Fig. 6*G*). Leptin levels were drastically increased in female *Adra1b*^LKO^ mice (Fig. 6*H*). Thus, liver *Adra1b* appears to be required for the metabolic adaptation to an obesogenic diet in female mice.

## DISCUSSION

Our understanding of how the brain regulates liver metabolism has significantly advanced since Claude Bernard (28) established in 1848 that pricking the floor of the fourth ventricle of the brain in rabbits resulted in increased hepatic glucose production. Since then, neuroanatomical work has described a profuse innervation in the liver of different species, revealing sympathetic and parasympathetic, afferent and efferent, circuits and connections (4, 5, 7, 8, 29–33). Functional evidence indicates that stimulation of the hepatic sympathetic nerves in rodents causes extensive changes in hepatic physiology and metabolism (4, 14, 34, 35). However, the identity of the adrenoceptors and pathways involved in these effects is still elusive. Here, we show that ADRA1B plays a role in mediating the effects of the SNS on hepatic metabolism, particularly in female mice.

Impaired glucose homeostasis in mice lacking *Adra1b* was previously reported (18). However, the use of a global knockout precluded to determine whether these effects were specifically due to its absence in the liver (18). In particular, targeted disruption of the mouse *Adra1b* gene causes cardiac defects and hypofertility (20–23), and *Adra1b* is highly expressed in the brain (19). Thus, the development and validation of a Cre-dependent model allowing targeted deletion of *Adra1b* provides a better appreciation of the contribution of liver ADRA1B signaling. Here, we demonstrate selective deletion in the liver after crossing *Adra1b*-flox and *Alb*-Cre mice, with no compensatory increase in other adrenoceptor subtypes. Despite being highly expressed in the liver, loss of hepatic *Adra1b* had limited impact in mice fed a laboratory chow diet. We speculate that the absence of phenotype could be attributed to either prenatal adaptations in the absence of *Adra1b* during the embryonic development of the liver or could underscore the importance of exposing mice to metabolic challenges to activate and study sympathetic outflow.

Although sex differences in the biochemistry of the liver have not been comprehensively studied to draw firm conclusions, some evidence suggests differences in hepatic glucose metabolism between sexes. For instance, females exhibit a greater ability of insulin to suppress hepatic glucose production (36–38). However, hyperinsulinemic-euglycemic clamp studies aimed at evaluating the effect of insulin on glucose disappearance rate yield no evidence of sex differences in the insulin-mediated state (37). Transcriptomics data suggest that the expression of receptors in liver of males and females differ (39), suggesting that the nature of the receptors involved in liver metabolism may differ between sexes. Here, we show that female, but not male, mice are more susceptible to obesity, glucose intolerance, and insulin resistance in the absence of liver *Adra1b*. It should be noted that despite impaired ITT, HOMA-IR was not different in obese female *Adra1b*^LKO^ mice. However, in contrast to humans, this index is generally not recommended in mice because it is poorly predictive of insulin sensitivity (40, 41). Thus, hyperinsulinemic-euglycemic clamp studies are warranted to better define the origin and sex differences of the insulin resistance that we observed. We also observed that females expressed higher levels of most adrenoceptors in the liver. Overall, the reasons for this sexual dimorphism are puzzling but may suggest an important synergy between sex hormones and the SNS for the control of liver metabolism. Additional work is needed to better understand this sex dimorphism.

Pair-fed female *Adra1b*^LKO^ mice did not exhibit a more drastic obese phenotype and had comparable glucose tolerance and insulin sensitivity to littermate controls. This indicates that the obesity is driving glucose impairments. Combined with the trend for increased food intake during metabolic cage experiments, this suggests that removing *Adra1b* from the liver may lead to a chronic and mild hyperphagia in female mice. How exactly such a manipulation in hepatocytes may influence feeding behavior is intriguing. One recent study reported that alterations in the liver molecular clock induce altered food patterns through afferent vagal signals (42). Whether impaired adrenergic signaling in the hepatocytes leads to similar feedback signals to the brain to influence circadian feeding is worth considerations.

Our results indicate that obese female mice lacking *Adra1b* in the liver have reduced gluconeogenic genes. Although this result may appear paradoxical at first glance, given that increased gluconeogenesis is a hallmark of liver insulin resistance, expression of *Pck1* was previously reported to be reduced in the livers of patients or mice with metabolic dysfunction-associated steatotic liver disease (MASLD) (43). In addition, this study revealed that liver *Pck1* deficiency aggravates liver fibrosis and inflammation. This raises the intriguing possibility that downregulation of *Pck1* in female *Adra1b*^LKO^ mice could lead to liver alterations in the long term, suggesting a potential protective role of liver ADRA1B in the progression toward MASLD. We also observed that female *Adra1b*^LKO^ mice fed an obesogenic diet had increased expression of *Cd36*, a fatty acid transporter whose expression is related to insulin resistance, inflammation, and steatosis (44, 45). Intriguingly, we observed slight but significant improvement in insulin sensitivity in lean mice fed laboratory chow. This observation is quite puzzling given the insulin resistance observed in obese female *Adra1b*^LKO^ mice but supports the bimodal hypothesis of the autonomic control of liver during metabolic diseases (6). In particular, this model predicts that obesity and type 2 diabetes may first lead to increased sympathetic outflow to the liver, followed by a progressive decrease in the number of hepatic sympathetic fibers and ultimately the development of hepatic neuropathy. In line with this model, it is plausible that the loss of liver *Adra1b* may be detrimental in the long term but not short term.

One interesting observation from our study is the downregulation of β-adrenoceptors in gWAT. Given the role of these receptors in mediating SNS-dependent lipolysis (46), this could explain the expansion of fat mass observed. In addition, activation of ADRB3 is well known to downregulate leptin expression and synthesis (46), which supports the observation that female *Adra1b*^LKO^ mice have increased *Lep* expression and circulating leptin levels associated with decreased *Adrb3* expression. Interestingly, global deletion of *Adra1b* was reported to lead to hyperleptinemia (18). Conversely, mice overexpressing constitutively active *Adra1a* and *Adra1b* globally were shown to have decreased leptin levels (47). Together, our data indicate that liver *Adra1b* could influence adipose lipid storage and endocrine functions in females.

One limitation of our study is that we did not measure liver or plasma levels of catecholamines. It was previously reported that liver noradrenaline levels were significantly higher in obese mice (48). Therefore, it is plausible that catecholamines increased as a compensation to the absence of liver *Adra1b*, which could have been even higher in combination with obesity. Nevertheless, our results clearly show that liver ADRA1B regulates systemic metabolism. Future studies are needed to exploit the hypothesis that this is consequent to a remodeling of the catecholaminergic system.

In conclusion, our study identified a role for liver ADRA1B in regulating energy and glucose metabolism. Our data highlight sex-dependent mechanisms by which the SNS regulates liver metabolism. Additional studies are needed to elucidate the mechanisms by which the ADRA1B receptor regulates liver metabolism, and especially why females are more likely to develop metabolic alterations in the absence of hepatic ADRA1B. A better understanding of the receptors and pathways involved in the sympathetic outflow of the liver will help develop a thoughtful perspective on how the autonomic control of peripheral organs is altered in metabolic diseases.

## DATA AVAILABILITY

Data will be made available upon reasonable request.

## SUPPLEMENTAL MATERIAL

10.6084/m9.figshare.26940505 Supplemental Table S1 and Fig. S1: https://doi.org/10.6084/m9.figshare.26940505.

## GRANTS

This work was supported by funding from the Canada Research Chairs Program (to A.C.), the Canadian Institutes of Health Research (CIHR) (to A.C.), the Natural Sciences and Engineering Research Council of Canada (NSERC) (to A.C), the Cardiometabolic health, Diabetes and Obesity Research Network (CMDO) (to A.C.), and the National Institute of Health (NIH) to J.K.E (R01DK118725, R01DK100659, P01DK119130) and to A.C. (K99DK120894). A.C. was supported by a Fonds de Recherche du Québec—Santé (FRQS) Research Scholar J1 award. A.S. was supported by a graduate scholarship from the FRQS. O.L. was supported by graduate scholarships from CIHR and FRQS.

## DISCLOSURES

No conflicts of interest, financial or otherwise, are declared by the authors.

## AUTHOR CONTRIBUTIONS

J.K.E. and A.C. conceived and designed research; A.S., M.M., O.L., S.B., and A.C. performed experiments; A.S., M.M., O.L., S.B., and A.C. analyzed data; A.S., O.L., S.B., and A.C. interpreted results of experiments; A.S., O.L., S.B., and A.C. prepared figures; A.S. and A.C. drafted manuscript; A.S., M.M., O.L., S.B., J.K.E., and A.C. edited and revised manuscript; A.S., M.M., O.L., S.B., J.K.E., and A.C. approved final version of manuscript.
